# GPs’ understanding of the benefits and harms of treatments for long-term conditions: an online survey

**DOI:** 10.3399/bjgpopen20X101016

**Published:** 2020-03-04

**Authors:** Julian Stephen Treadwell, Geoff Wong, Coral Milburn-Curtis, Benjamin Feakins, Trisha Greenhalgh

**Affiliations:** 1 NIHR Doctoral Research Fellow, Nuffield Department of Primary Care Health Sciences, University of Oxford, Oxford, UK; 2 Clinical Research Fellow, Nuffield Department of Primary Care Health Sciences, University of Oxford, Oxford, UK; 3 Associate Fellow, Green Templeton College, University of Oxford, Oxford, UK; 4 Biostatistician, Nuffield Department of Population Health, University of Oxford, Oxford, UK; 5 Professor of Primary Health Care, Nuffield Department of Primary Care Health Sciences, University of Oxford, Oxford, UK

**Keywords:** prescribing, family medicine, comorbidity, long-term care, clinical decision-making

## Abstract

**Background:**

GPs prescribe multiple long-term treatments to their patients. For shared clinical decision-making, understanding of the absolute benefits and harms of individual treatments is needed. International evidence shows that doctors’ knowledge of treatment effects is poor but, to the authors knowledge, this has not been researched among GPs in the UK.

**Aim:**

To measure the level and range of the quantitative understanding of the benefits and harms of treatments for common long-term conditions (LTCs) among GPs.

**Design & setting:**

An online cross-sectional survey was distributed to GPs in the UK.

**Method:**

Participants were asked to estimate the percentage absolute risk reduction or increase conferred by 13 interventions across 10 LTCs on 17 important outcomes. Responses were collated and presented in a novel graphic format to allow detailed visualisation of the findings. Descriptive statistical analysis was performed.

**Results:**

A total of 443 responders were included in the analysis. Most demonstrated poor (and in some cases very poor) knowledge of the absolute benefits and harms of treatments. Overall, an average of 10.9% of responses were correct allowing for ±1% margin in absolute risk estimates and 23.3% allowing a ±3% margin. Eighty-seven point seven per cent of responses overestimated and 8.9% of responses underestimated treatment effects. There was no tendency to differentially overestimate benefits and underestimate harms. Sixty-four point eight per cent of GPs self-reported ‘low’ to ‘very low’ confidence in their knowledge.

**Conclusion:**

GPs’ knowledge of the absolute benefits and harms of treatments is poor, with inaccuracies of a magnitude likely to meaningfully affect clinical decision-making and impede conversations with patients regarding treatment choices.

## How this fits in

Clinicians’ understanding of the benefits and harms of medical interventions has been shown to be poor in a variety of clinical settings and countries, with a tendency to overestimate benefits and underestimate harms. This has not been studied among GPs in the UK. This study found wide-ranging inaccuracies in GPs’ estimates of the benefits and harms of treatments with overestimation of both benefits and harms predominating. This is important in the UK, where GPs play a critical role in the management of LTCs.

## Introduction

In the UK, GPs are responsible for the majority of care for patients’ LTCs, such as hypertension, diabetes, and osteoporosis.^1^ With GPs supported by clinical guidelines and encouraged by performance indicators,^2^ this usually results in the prescription of long-term medication aimed at reducing the risk of adverse outcomes. As the prevalence of multimorbidity has increased,^3^ so too have levels of polypharmacy, which may cause harm in addition to the treatments’ intended benefits.^4,5^ The resulting burden of care for patients can be high.^6^

Guidance on the management of multimorbidity from the National Institute for Health and Care Excellence (NICE) contains a key recommendation that clinicians should: *'* *Review medicines and other treatments taking into account evidence of likely benefits and harms for the individual patient and outcomes important to the person* *.’* ^7^

If GPs are to do this, they require an understanding of the absolute chance of benefits or harms conferred by treatments; however, research shows that clinicians have poor knowledge of this kind of information. A systematic review by Hoffmann and Del Mar, including 48 studies involving clinicians from a range of disciplines and countries, revealed marked inaccuracies in their understanding, with a tendency to overestimate benefits and underestimate harms. Only five of these studies involved GPs and none involved GPs in the UK.^8^

Therefore, the present study sought to answer the question: what is the degree of knowledge among GPs in the UK regarding the effect size of the benefits and harms of treatments for common LTCs?

## Method

An online cross-sectional questionnaire survey aimed to assess the level and range of GPs’ quantitative understanding of the benefits and harms of treatments for common LTCs.

### Questionnaire development

The method described by Burns *et al* was employed.^9^ A long list of questions was generated based on common clinical scenarios in primary care for which clinical guidelines recommend ≥1 long-term treatments. The present study sought to address a number of domains of interest: treatment benefits and harms, treatments with an effect on surrogate outcomes, and those with an effect on composite outcomes. The present study also explored knowledge about relatively high and relatively lower value treatments. An advisory panel of three topic experts assisted with a process of item reduction to select 20 stem questions. These were tested at face-to-face pre-testing meetings with two groups of four GPs, leading to adjustments to language, order, and presentation. Online pilot testing was conducted with 29 other GPs. The advisory panel conducted clinical sensibility testing of the final questionnaire to assess its comprehensiveness, clarity, and face-validity. The survey was hosted on SurveyMonkey.

### Question design

**Figure 1.**
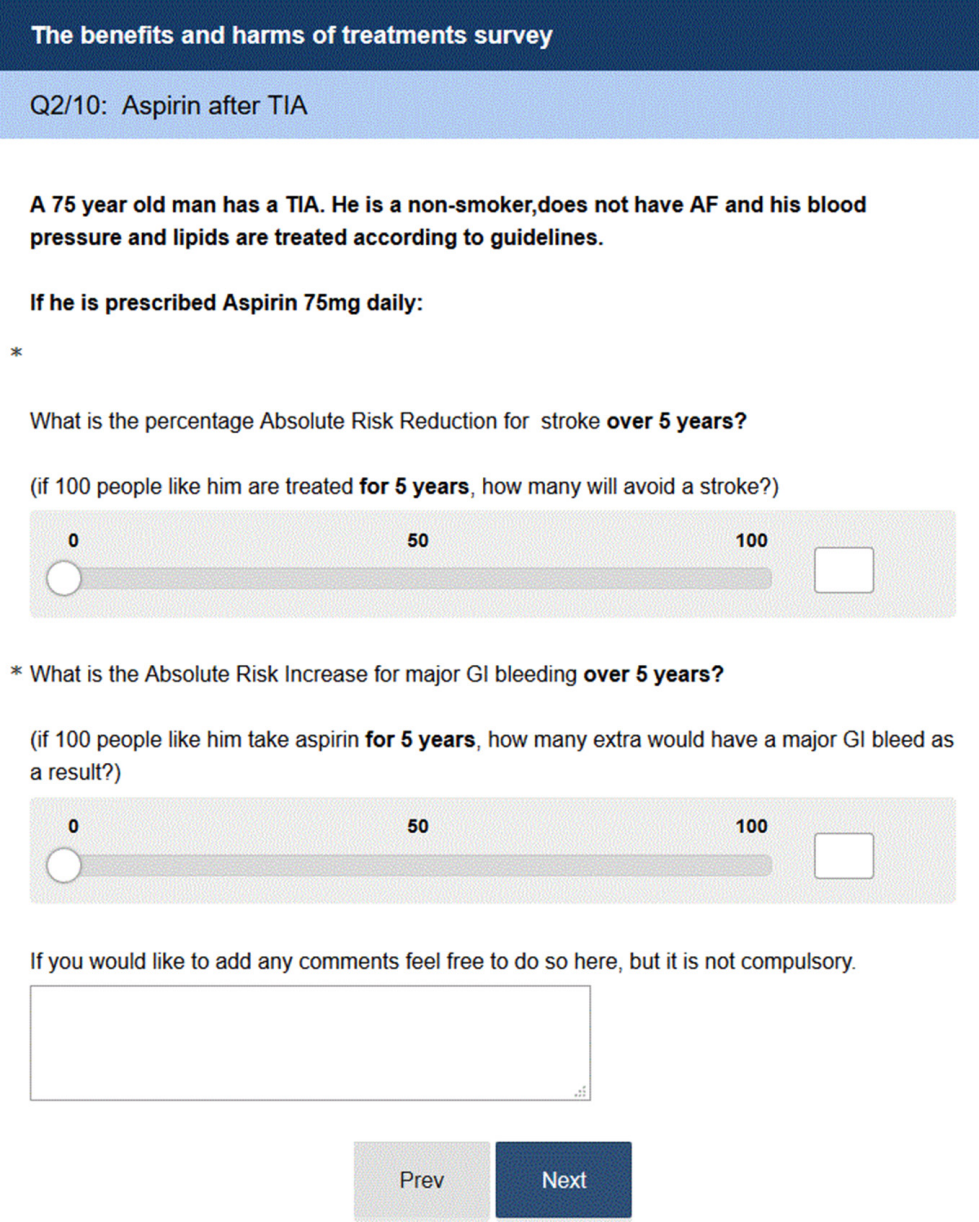
Example survey question. AF = atrial fibrillation. GI = gastrointestinal. TIA = transient ischaemic attack.

Each question had a stem describing a fictional patient with an LTC for whom current guidelines recommend a treatment. Participants were asked to estimate the absolute risk reduction (or increase) in various outcomes conferred over a defined time period. An example question is shown in Figure 1.

The fictional patient's characteristics were aligned with average participant characteristics from key clinical trials or systematic reviews in order to define a 'correct' evidence-based answer. Participants were asked to give their 'best estimate or rough idea', acknowledging that few would be expected to know the answers precisely. To overcome misunderstanding of the term 'absolute risk reduction', an explanation was provided. In addition, each question included a re-wording in natural frequency format (for example, 'If 100 people like him are treated for 5 years, how many will avoid a stroke?'), which has been shown to improve understanding of risk in patients and clinicians.^10^ All questions, with the source of the evidence-based 'correct' answers, are in Supplementary Table S1.

Pre-testing showed that responders would be unlikely to complete more than 10 stem questions, so two groups of 10 were created: survey one and two. Question order and placement were decided in pre-testing to encourage survey completion. Each question was presented on a new screen. Responses were compulsory: failing to give a response blocked progress to the next question and led to survey drop-out. This strategy (as opposed to non-compulsory answers) was judged to be most likely to maximise total question response rates, as temptation to leave out challenging questions while continuing onto the next might be high. Responders were able to navigate to previous questions and amend answers.

Responders were asked to rate their confidence in their answers on a 5-point Likert scale and completed an electronic consent page at the start of the survey.

### Survey distribution

The inclusion criterion was GPs currently practising in the UK. Exclusion criteria were GPs in training and GPs not in clinical practice within the last 3 years. Responders confirmed these electronically.

Invitations were distributed by direct email or in an email bulletin from 14 English clinical commissioning groups, one Scottish health board, one Welsh local health board, the National Association for Sessional GPs, one regional GP education group, the Royal College of General Practitioners Research Ready network, and two Facebook groups. Some invitations may have been forwarded further. These routes were chosen to reach an undifferentiated pool of GPs to create a sample frame representative of the GP population. Because of this open approach, the total number of GPs who received an invitation could not be established.

Survey one and two were distributed using a pragmatic approach by estimating the number of recipients in email lists to balance the distribution of each survey, adjusting over the course of the study by response rates. Therefore, each survey had a different sample of responders.

Reminder emails were sent at 2 and 4 weeks. Participants were invited to enter a prize draw for an iPad mini.

Data handling complied with the General Data Protection Act. The University of Oxford was the data controller for purposes of the Act.

### Analysis

For each question, a figure for the average inaccuracy of estimates (distance from the correct answer) was calculated by subtracting each score from its corresponding correct score and recording as an absolute difference. A mean was then calculated.

The percentage of responders giving a correct answer was calculated using two definitions of 'correct'. First, a narrow definition allowing only ±1% absolute risk estimate outside the correct answer calculated to the nearest integer. Second, a wider definition allowing a ±3% margin. These ranges were chosen after expert panel discussion; although unavoidably arbitrary, they were felt to be reflective of the importance of accuracy of estimates in clinical practice.

The percentage of responders under- or over-estimating the treatment effects was calculated. For this, a correct answer was defined as responders estimating correct to the nearest integer.

For a UK population of 59 597 GPs,^11^ with a confidence level of 95% and an average confidence interval (CI) of 5, a sample size of 382 was required. To assess whether withdrawal might cause any systematic bias, Little's missing completely at random (MCAR) test for missingness was applied.^12^

Between-group differences in response accuracy were analysed using *t*-tests (for example, sex groups) and analysis of variance (for example, differences across age groups, geographical location, and GP roles). Correlational analysis explored relationships between levels of confidence and competence. Significance levels were set at 5%.

## Results

The survey was distributed from 5 June to 4 September 2018. A total of 511 responses were received: 229 for survey one and 282 for survey two. They had completion rates of 72.9% and 68.4%, respectively. Sixty-eight (13.3%) participants withdrew without answering a single clinical question, leaving 443 responders in the analysis. Answers to clinical questions by responders who later withdrew were included.

Demographic details of responders are shown in Table 1. The sample was broadly representative of GP roles, age, and sex, with wide geographical spread.

**Table 1.** Demographic characteristics of survey responders (*N* = 511)

**Characteristic**	**Responders, % (*n***)
**Clinical** **r** **ole** **:** GP principal	53.0 (271)
Salaried GP	26.8 (137)
Locum GP	16.6 (85)
Retainer GP	1.2 (6)
Other	2.3 (12)
**Sex** **:** Female	66.7 (341)
Male	32.9 (168)
Other/prefer not to say	0.4 (2)
**Age, years:** <30	0.6 (3)
30–39	28.6 (146)
40–49	35.0 (179)
50–59	28.6 (146)
≥60	7.2 (37)
**Region:** South West England	21.7 (111)
Greater London	6.8 (35)
South East England	13.5 (69)
East of England	8.0 (41)
East Midlands	3.5 (18)
West Midlands	5.1 (26)
North West England	6.7 (34)
North East England	4.1 (21)
Yorkshire and Humber	4.1 (21)
Northern Ireland	0.8 (4)
Wales	9.4 (48)
Scotland	16.2 (83)

A response rate could not be calculated owing to the absence of a distribution denominator, as described in the Method section.

Little’s MCAR test for missingness estimated that both surveys produced negligible levels of missingness. For example, survey one: Little's MCAR: χ^2^ (79) = 71.969; **P** *=* 0.700. For survey two: Little's MCAR: χ^2^ (61) = 66.259; **P** *=* 0.300.​

### Visualisation of responses per question

**Figure 2.**
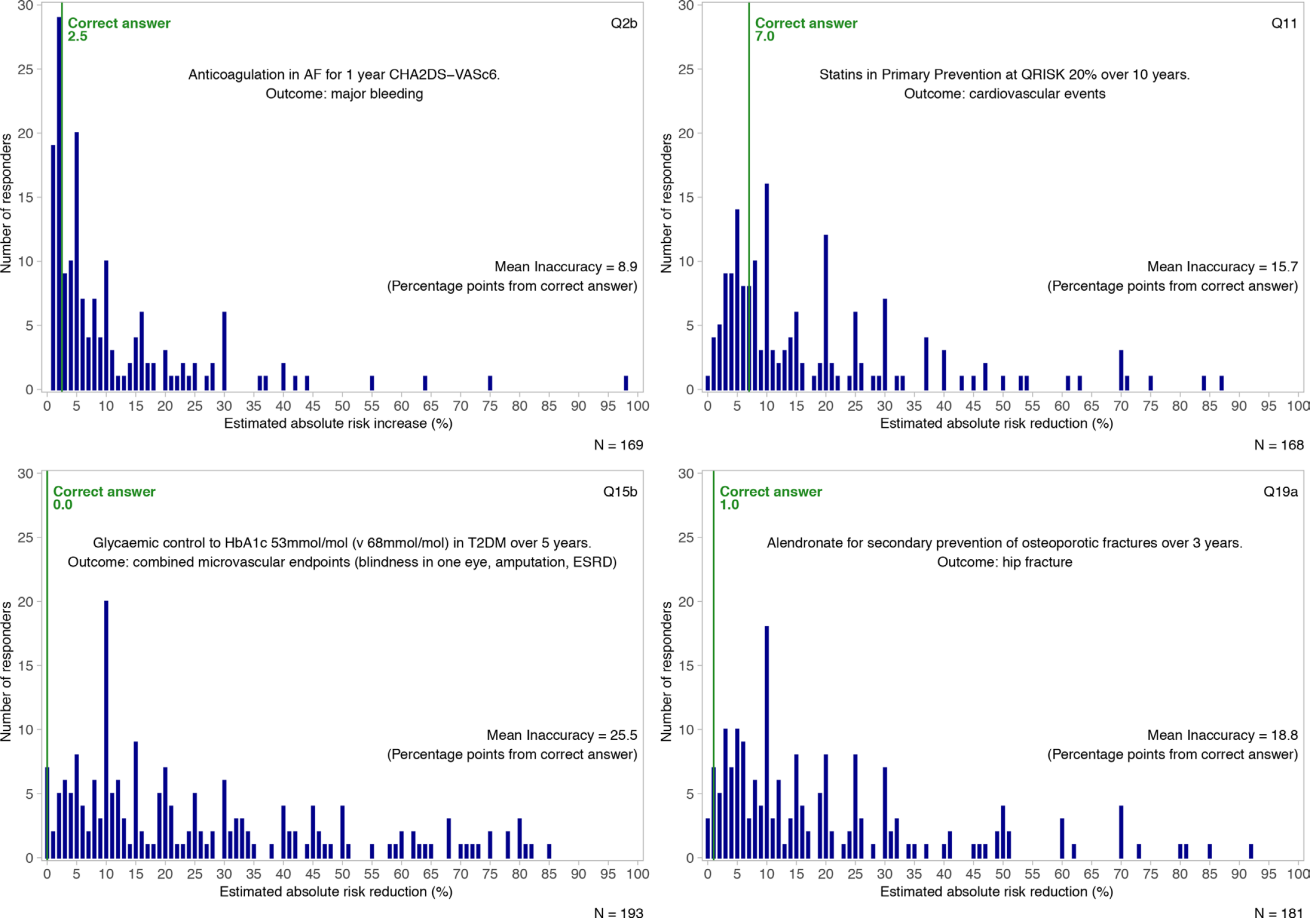
Responses to four survey questions. AF = atrial fibrillation. ESRD = end-stage renal disease. T2DM = type 2 diabetes mellitus.

**Figure 3.**
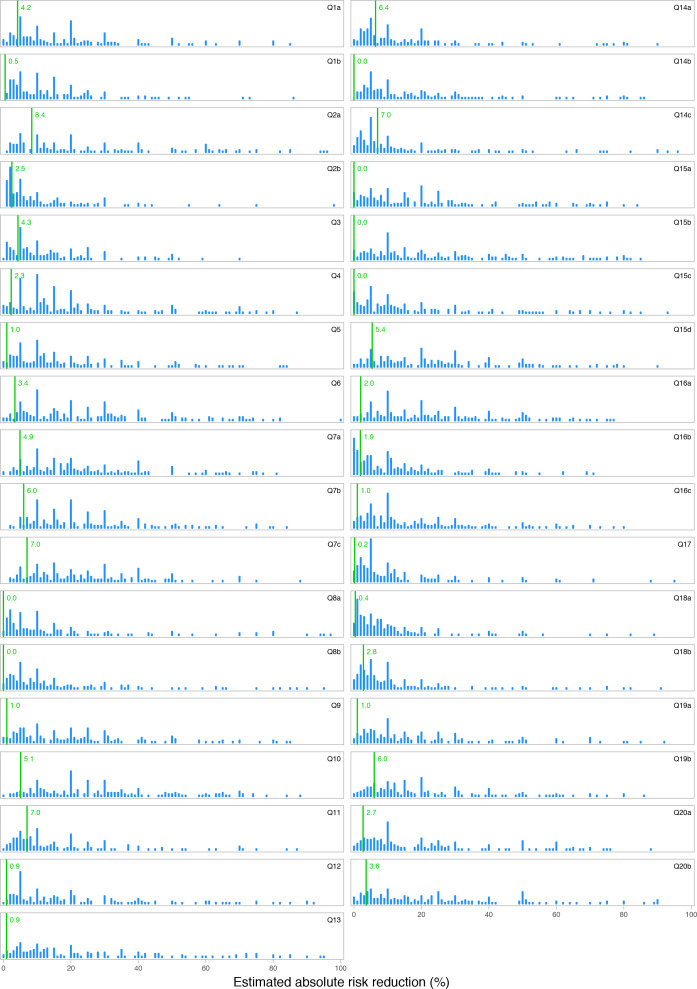
All survey question responses collated graphically. Green line = correct answer.

Figure 2 illustrates the responses for a selection of four questions alongside the correct evidence-based answer. A summary visualisation of all 35 answers is presented in Figure 3, with detailed versions available in Supplementary Figure S1. There is a very broad spread of estimates, with the majority of responses some distance away from the correct answer.

### Mean inaccuracy per question

Figures for the mean inaccuracy per question are included in Figure 2 and Supplementary Figure S1, ranging from 8.9 (Q2b: major bleeding risk with anticoagulation), to 25.5 (Q15b: the effect of tight glycaemic control on diabetic microvascular outcomes). The mean level of inaccuracy for all questions was a 17.5% absolute difference between estimates and correct answers. The mean correct absolute risk reduction or increase of the survey questions was 2.9%.

### Proportions of correct responses per question

**Figure 4.**
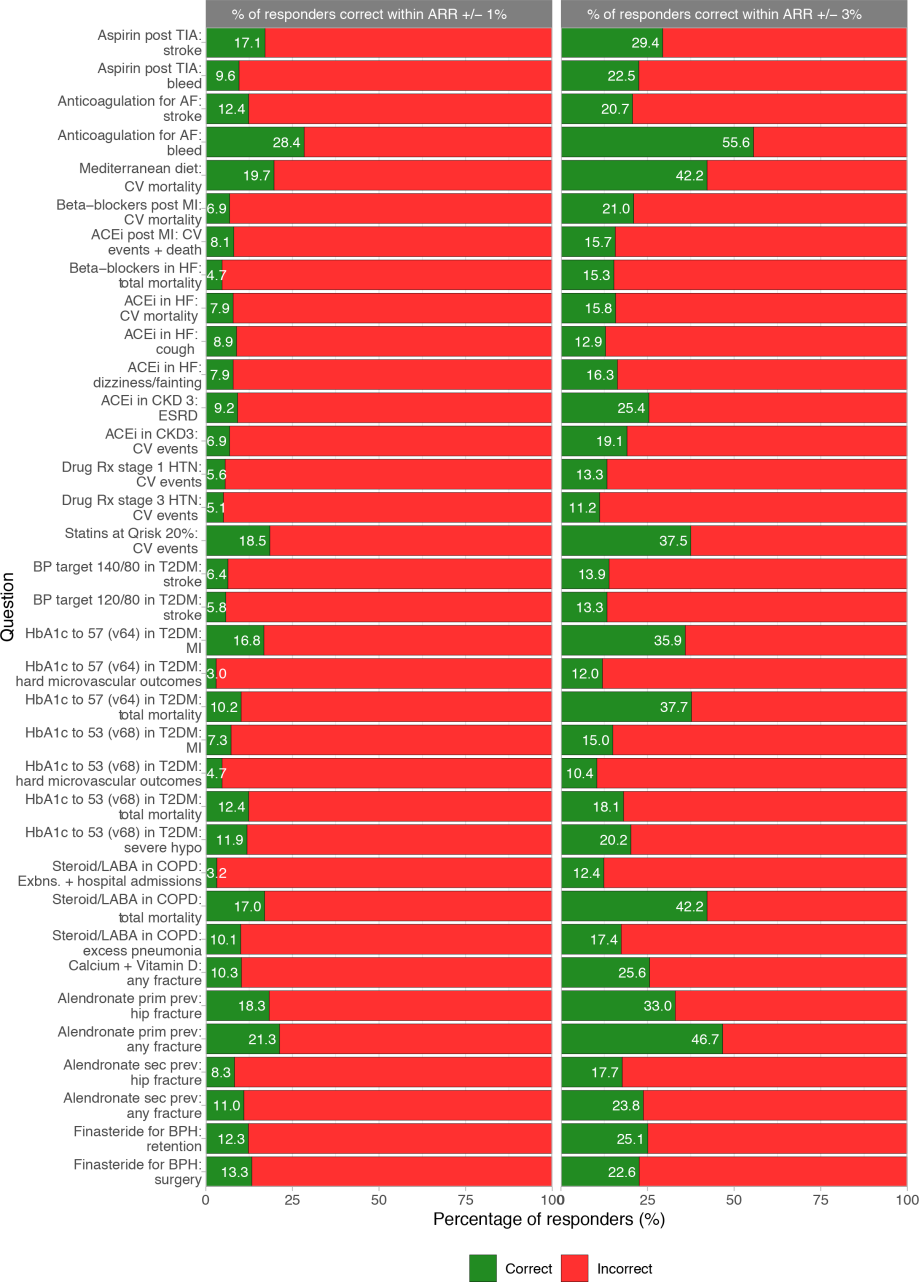
Percentage of correct responses per survey question. ACEi = angiotensin-converting enzyme inhibitors. AF = atrial fibrillation. ARR = absolute risk reduction. BP = blood pressure. BPH = benign prostatic hyperplasia. CKD = chronic kidney disease. COPD = chronic obstructive pulmonary disease. CV = cardiovascular. ESRD = end-stage renal disease. Exbns = Exacerbations. HF = heart failure. HTN = hypertension. hypo = hypoglycaemia. LABA = long-acting Beta 2 agonists. MI = myocardial infarction. prim prev = primary prevention. Rx = Treatment. Sec prev = secondary prevention. T2DM = type 2 diabetes mellitus. TIA = transient ischaemic attack.

The percentage of responders giving a correct answer for each question are illustrated in Figure 4. The proportion ranged from 3.0%–28.4% (mean = 10.9%) and 10.4%–55.6% (mean = 23.3%) using the narrow and wide definitions of correct, respectively.

### Over- versus under-estimation of absolute risk reduction or increase

There was a strong tendency to overestimate treatment effects, both benefits and harms. For all questions combined, 87.7% of responders overestimated and 8.9% underestimated treatment effects.

### Between-group differences in accuracy of responses

No meaningful differences in response accuracy were found by age, sex, geographical location, or GP role.

### GPs’ self-rated confidence

**Figure 5.**
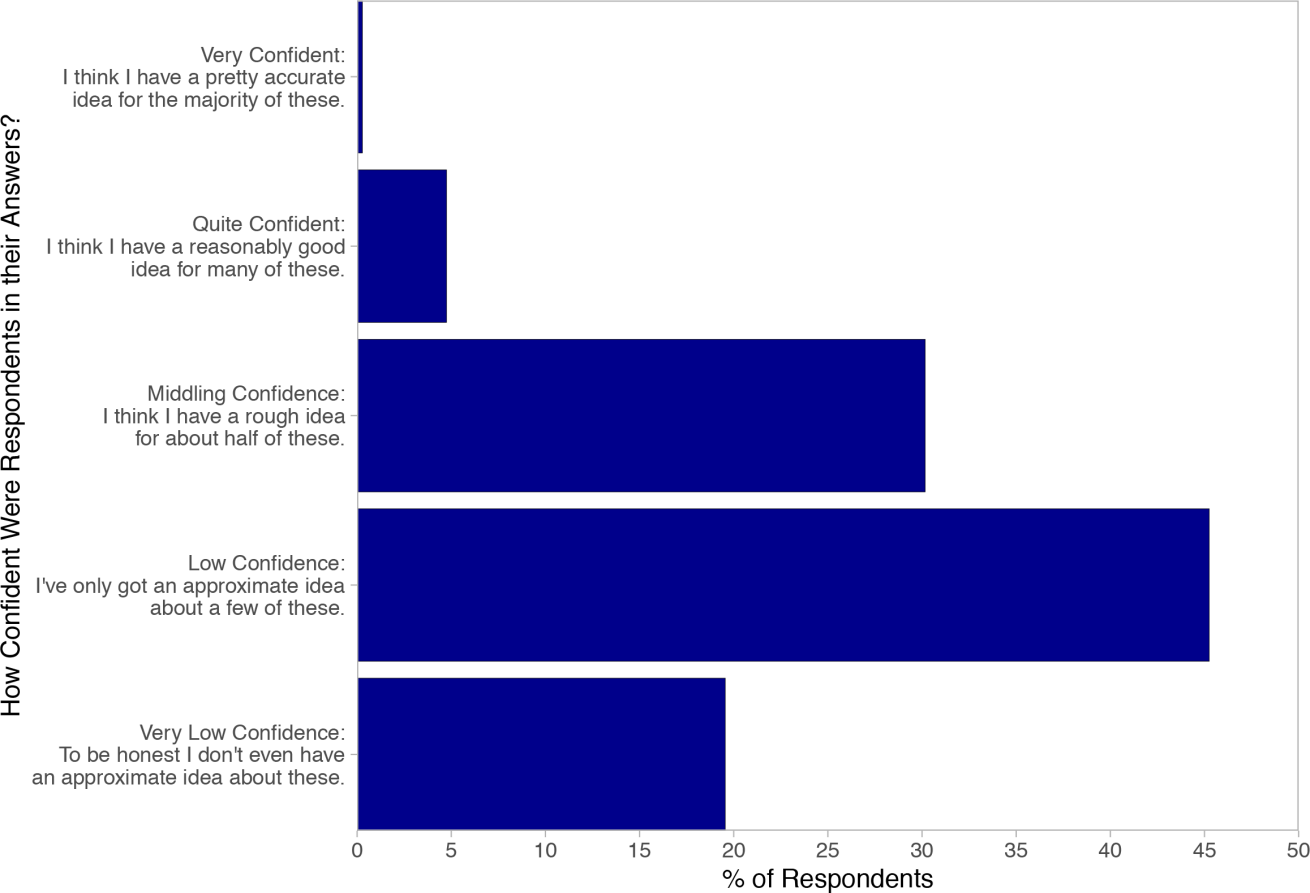
Survey responders’ confidence in their answers.

Figure 5 shows GPs’ self-rated confidence in their answers. They declared poor overall confidence, with 64.8% reporting ‘low’ or ‘very low’ confidence and only 5.0% reporting they were ‘quite’ or ‘very’ confident. There was a statistically significant correlation between self-reported confidence and accuracy of responses, but confidence only predicted a small amount of the variation in answers (*r* = 0.275; **P**<0.001; *n* = 405).

## Discussion

### Summary

The present study shows inaccuracies in GPs’ knowledge of the absolute benefits and harms of treatments of a magnitude likely to meaningfully affect clinical decision-making and impede conversations with patients regarding treatment choices. This is important given the key role GPs have in managing LTCs for their patient populations.

### Strengths and limitations

The survey was conducted using best methodological guidance^9,13–16^ to develop a high-quality questionnaire with low risk of measurement error. Coverage error was minimised by using a careful sample frame of undifferentiated GPs.

The findings are presented using detailed graphics in addition to summary analyses, allowing a fuller understanding of the responses than previous studies.

The final sample of 443 was large enough to address population external validity and thus potential generalisability of the results.

A minor limitation is a lack of information on distribution numbers, rendering it impossible to calculate a response rate; however, the representativeness of the sample frame supports a reliable set of responses.

There will inevitably be some non-response error, comprising total non-responders and partial non-response from drop-out. Both of these would be expected to bias the results towards more accurate answers, as doctors with confidence in the subject would be more likely to respond. Therefore, the results might overestimate the accuracy of GP knowledge.

### Comparison with existing literature

In contrast to the review by Hoffman and Del Mar,^8^ who found a tendency for clinicians to overestimate benefits and underestimate harms, the present study found an overestimation of both benefits and harms. A possible cause of this may be the unique perspective of GPs, who are more likely to witness harms and side effects of medications owing to continuity of care and their accessibility to patients.

Only five studies identified in the systematic review by Hoffman and Del Mar^8^ included GPs;^17–21^ these only addressed single conditions (sample sizes 42–525). All found poor understanding of quantitative effects.

Surveys involving specialists gave similar findings. Physicians overestimated the benefits of interventions in type 2 diabetes by as much as 30%.^22^ Ninety per cent of liver transplant surgeons overestimated the chance of graft survival at 3 years.^23^ Forty-three per cent of paediatricians overestimated the ability of antibiotics to prevent complications of otitis media or tonsillitis.^24^

To the authors’ knowledge, the present study is the first conducted with UK GPs and the first anywhere to address such a broad range of clinical practice.

### Implications for practice and research

Dependent on clinical context and patient preferences, these inaccuracies in understanding could have negative implications for shared decision-making. For example, imagine a patient at low cardiovascular risk wishing to discuss whether to take drug treatment for stage 1 hypertension (Q9): the conversation will be very different if the patient talks to a doctor who understands the benefit to be a 1% absolute risk reduction in cardiovascular events over 10 years rather than one who thinks it is 20%. Consider also how a doctor might encourage a patient with type 2 diabetes to take treatment for tight glycaemic control if they believe it will reduce the chance of hard microvascular outcomes by 10% over 5 years (as 20% of responders did in Q15b) rather than 0% (what the key trial showed). Underestimation by clinicians of the benefits of anticoagulant therapy for stroke prevention in atrial fibrillation coupled with an overestimation of bleeding risk is known to be a factor in the under-use of this highly effective treatment.^25,26^

It is no surprise that most GPs did not know the correct answers. The authors would not expect any individual to remember a high volume of precise figures. What is concerning is the degree of misestimation of treatment effects, usually overestimation.

This is perceived as a system issue rather than a failure of individual learning. Quantitative information about the benefits and harms of treatments is difficult and time-consuming to find. It rarely features in clinical guidelines^27^ and clinicians would need to read original scientific articles, which they have neither the time^28^ nor expertise^29^ to do. Some resources exist online,^30–32^ but are varied in location and not comprehensive in content.

The very poor levels of confidence reported by responders is discomforting. GPs wish to provide individualised care for their patients and have identified the rigid application of guidelines as a driver for inappropriate or harmful treatment.^33,34^ Guidelines are not tramlines,^35^ but without access to this vital quantitative information, applying clinical judgment and responding to patients’ preferences is very challenging. Research has not focused on GPs' attitudes to this particular knowledge deficit, but it may well have an effect on job satisfaction: 'tick-box' and target-driven cultures are recognised contributors to burnout.^36^

Remedies to this problem include improved guidelines and information resources to provide accessible and understandable quantitative information on both the benefits and harms of treatments. The authors are currently working on a new resource for GPs (https://fundingawards.nihr.ac.uk/award/DRF-2018-11-ST2-021), focusing on usability and relevance in practice; however, information resources alone are unlikely to be enough. They will need to be accompanied by further research and measures to address the understanding and communication of risk in the consulting room, in addition to changes in the structural and cultural drivers that influence clinical practice. Following this, the best available evidence may be able to be combined with clinical judgment and patient preference, which is the original goal of evidence-based medicine.^37^
